# A Case of Rotator Cuff Tear Owing to External Electrical Cardioversion

**DOI:** 10.1002/joa3.70412

**Published:** 2026-07-02

**Authors:** Masahiro Watanabe, Shinichi Higuchi, Ayano Yuasa, Syun Hasegawa, Kinichi Kameyama

**Affiliations:** ^1^ Tokyo Metropolitan Tama‐Hokubu Medical Center Higashimurayama Japan; ^2^ Tokyo Women's Medical University Shinjuku City Japan

**Keywords:** atrial fibrillation, electrical cardioversion, rotator cuff tear

## Abstract

External defibrillation during ablation complicated a massive rotator cuff tear.

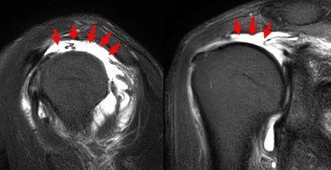

A 77‐year‐old man, who visited the outpatient clinic following the detection of an irregular pulse during a medical checkup, was diagnosed with atrial fibrillation. Due to persistent shortness of breath, he opted for radical treatment and was admitted to the hospital for catheter ablation. The patient had no prior history of orthopedic conditions or shoulder joint dysfunction. During the ablation procedure, the patient was placed in a supine position with both wrist and knee joints secured by restraints. Sedation was managed using noninvasive positive pressure ventilation in combination with dexmedetomidine and fentanyl; the Bispectral Index was carefully maintained at 70 or below to ensure a consistent level of light sedation. Because atrial fibrillation persisted during the procedure, synchronized external cardioversion (100 J) was performed by attaching pads to the right front and left sides of the chest. The patient returned to sinus rhythm, and the procedure was completed successfully after confirming the isolation of the pulmonary veins and the superior vena cava. Although the patient reported no shoulder pain immediately post‐procedure, he began complaining of pain during shoulder movement on the first postoperative day, accompanied by a limited range of motion in the right shoulder joint. Initial radiographic findings showed no signs of bone injury or fracture. However, because the pain during movement persisted for 1 month, magnetic resonance imaging was performed, revealing an extensive right shoulder rotator cuff tear involving the supraspinatus, subscapularis, and infraspinatus tendons with hemarthrosis (Figure 1). The patient had no prior history of shoulder joint pain or dysfunction, and there were no episodes of trauma, such as a fall, between the procedure and the onset of symptoms. MRI also showed almost no muscle atrophy and it was considered that the rotator cuff tear was acute. Consequently, the injury was diagnosed as a rotator cuff tear resulting from the cardioversion. Two months later, despite persisting pain, his range of motion had improved. Surgery was considered; however, the patient opted for conservative treatment. Four months later, his shoulder pain had almost fully resolved.

**FIGURE 1:**
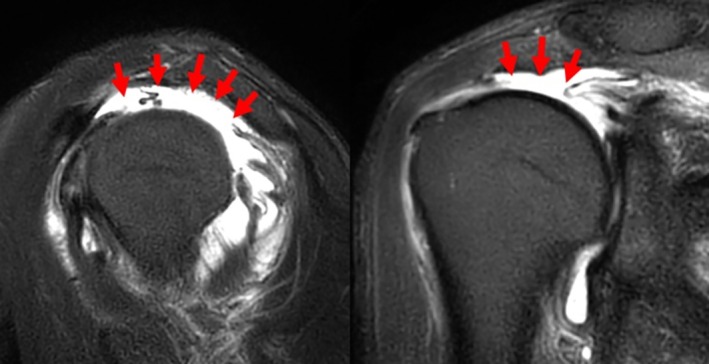
Magnetic resonance imaging at diagnosis showing a right rotator cuff tear (arrow).

This case illustrates a significant musculoskeletal complication from a common electrophysiological intervention.

While external cardioversion is generally associated with complications like arrhythmia or skin burns, the forced muscle contraction caused by electrical stimulation can lead to musculoskeletal injury [1]. The prevalence of rotator cuff tears increases significantly with age, reaching approximately 45% in individuals aged 70–79 as tendons undergo degenerative changes that increase susceptibility to even minor trauma [2]. In this instance, two factors likely converged: the patient's age‐related vulnerability and the physical restraint of the wrists. While the surgical drapes prevented direct visualization of the patient's upper limb movement during the shock, the wrists remained firmly restrained as shown in the author's replication (Figure 2). The restraint may have acted as a fulcrum, preventing the energy of the sudden, violent muscle contraction from being dissipated through limb movement and instead concentrating the stress on the rotator cuff muscles (supraspinatus, infraspinatus, teres minor, and subscapularis). Similar musculoskeletal complications, such as shoulder dislocations and tendon tears, have been reported following defibrillation for ventricular tachycardia or testing for subcutaneous implantable cardioverter‐defibrillators (S‐ICDs) [3, 4].

**FIGURE 2:**
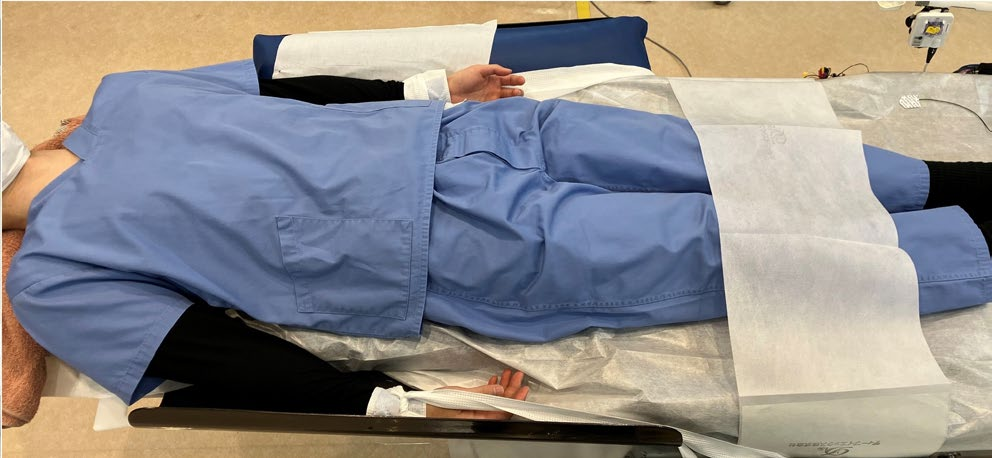
Restraint during ablation (author's replication).

It is unclear whether rotator cuff tears are caused by excessive extension of the upper limb during defibrillation or by excessive contraction of the rotator cuff muscles. Shoulder joint restraint may prevent excessive extension during external defibrillation. Additionally, selection of intracardiac defibrillation, which requires a lower energy threshold, is considered a potential factor for reducing the risk of onset.

Ultimately, if a patient experiences new‐onset pain during shoulder movement or mobility issues following cardioversion, a shoulder joint injury should be strongly considered in the differential diagnosis to ensure timely orthopedic intervention.

## Consent

Written informed consent for the submission and publication of this report was obtained from the patient.

## Conflicts of Interest

The authors declare no conflicts of interest.

## Data Availability

The data that support the findings of this study are available from the corresponding author upon reasonable request.
